# Appendicitis: From the Standpoint of a Country Doctor

**Published:** 1899-07

**Authors:** A. C. Davidson

**Affiliations:** Sharon, Ga.


ATLANTA

### Journal-Record of Medicine.

Successor to Atlanta Medical and Surgical Journal, Established 1855, and Southern Medical Record, Established 1870.

VOL. I. JULY, 1899. No. 4.

BERNARD WOLFF, M.D., DUNBAR ROY, A.B., M.D., EDITORS. M. B. HUTCHINS, M.D., BUSINESS MANAGER.

PUBLISHED MONTHLY. OFFICE 305 AND 306 FITTEN BUILDING.

### ORIGINAL COMMUNICATIONS.

### APPENDICITIS: FROM THE STANDPOINT OF A COUNTRY DOCTOR.*

BY A. C. DAVIDSON, M.D.,

SHARON, GA.

In presenting this paper to this intelligent and learned body of physicians and surgeons, I do not for one moment presume that any new or more interesting phase may be presented than that which has already been more elegantly written and more eloquently said about this much-talked-of and much-written-about and much-dreaded disease.

However, it is not so much with regard to the diagnosis or general pathology or with the treatment of this disease that this paper shall have to do, but it is more of a presentation of some general

*Read before the Georgia Medical Association, in Macon, Ga.

observations and deductions with regard to the appendix itself; and with regard to the frequency, or rather with regard to the infrequency of appendicitis as compared with the frequency of other diseases involving organs of like textural anatomy; and a presentation of some reasons why the said infrequency obtains, and of some further observations and facts, which, could they be more generally disseminated among the laity, would go far to prevent and cure that somewhat morbid mental condition which prevails to a considerable extent among the more intelligent and better classes, which for the want of a better name I have denominated appendicitiphobia.

The vermiform process is a tube-like cul-de-sac which arises from the fundus of the cecum at the juncture of the longitudinal muscular bands which pass over the cecum and head of the colon in common with the rest of the large intestines.

It varies in length from one to four inches; its average diameter is about two lines, or the size of a No. 8 catheter, American gauge, and it is somewhat larger at its terminal or closed end. It lies somewhat in the same direction but not quite parallel with the ileum, and both enter from the same direction into the same side of the cecum, and it is sometimes provided with an imperfect valve at its open end.

It is said to be rudimentary, and is supposed to be functionless and useless. This opinion obtains among those who accept the theory of evolution as a solution of the mystery of the origin and development of all animal life. Holding to this theory, they suppose that the vermiform process is yet in its rude and undeveloped state, or just as it was when the entire human body had just come into its being, and was new and rude, and, according to their theory, was not at all possessed of its present stature, symmetry, beauty and grace, and when forsooth it had need of an appendix or elongated cecum. These opinions will be noticed again as we proceed.

The textural anatomy of the appendix does not differ materially from that of the cecum, of which it is a part. It is composed of mucous, submucous, muscular and serous membranes similar to those of the other parts of the intestinal tube. Its glandular and lymphatic structures are not unlike those of the large intestines, and its connective and areolar tissues do not differ from similar textures in other organs of the human body.

It is not, as some anatomists assert, the true base of the cecum, but is situated somewhat above, and behind the real bas-fond of that organ.

Aside from the fact that it is a blind tubelike cul-de-sac, there is no reason why it should be more subject to inflammatory action than any other organic structure of similar component parts; and, notwithstanding it is a blind, wormlike tube, and having its open end situated near the base of the cecum, and therefore supposed to be liable to become the receptacle of various kinds of indigestible ingesta which enter with food substances, and otherwise, the alimentary canal, yet it is a fact that it is not more subject to inflammatory or other diseased action than are other organic structures of similar constituency.

This is the observation of a country doctor with regard to country districts.

During a period of twenty-two years, in which I have been actively engaged in a continuous and somewhat considerable practice, extending into more than fifteen counties, and having during that period of time treated more than three thousand different persons sick of the various diseases to which human flesh is heir, I have been brought in touch with only seven cases of what was supposed to be well developed cases of appendicitis.

Two of the seven were cases to which I was called as consultant. Five were my own cases, and of these five cases three were in the same man, occurring, however, at intervals of more than three years between attacks.

Thus it appears, and correctly, that in my own individual practice, during the time above written, I have been associated professionally with only three persons afflicted with appendicitis in a clientele of more than three thousand.

The exceeding small percentage of appendicular diseases occurring in my practice is not a peculiar nor exceptional experience, but obtains among all my rural confrères of whom I have sought statistical data.

Now, without taking cognizance of such inflammatory diseases as pleuritis, pneumonitis, bronchitis, laryngitis, pericarditis and such other inflammatory diseases as are peculiar to the thoracic region, or of such diseases as pelvic cellulitis, salpingitis, cystitis and other inflammatory diseases peculiar to the region of the pelvis; all diseases of organs whose textural anatomy is somewhat, if not altogether, similar to the anatomic structure of the appendix; and taking notice only of such inflammatory diseases as gastritis, enteric and gastro-enteric fever, ileo-colitis, proctitis and other diseases peculiar to the alimentary canal, diseased conditions of organs whose anatomic structure is not only somewhat but altogether similar to the anatomic structure of the appendix, we find—in the country districts where thousands and tens of thousands of raspberries, blackberries, tomatoes, muscadines and grapes of all kinds, wild and cultivated, and other fruits abound, and which are eaten by the inhabitants indiscriminately and without restriction, regardless of the millions of seeds the said fruits contain—that these last-named inflammatory diseases occur more than fiftyfold more frequently than does appendicitis.

Now, should these data taken from my own individual experience and from the experience of my rural medical confrères be objected to upon the ground of a possible inaccuracy of diagnosis, then from this objection to these experiences I appeal to the vital statistics of the great cities.

Taking Philadelphia as a fairly representative city and its vital statistics as a fair sample of the vital statistics of the other great cities, and taking it for granted that these statistics are correct, and that those physicians from whose reports the aforesaid vital statistics were tabulated made no mistake with regard to diagnosis, and that a greater per cent. of cases of appendicitis recovered in their hands than recovered from any other diseases treated, yet by reference to these same statistics as they appear from week to week in that very much up-to-date medical weekly, the *Philadelphia Medical Journal*, we find that not many more deaths from appendicitis have occurred in the thousand inhabitants of the great cities than have occurred in the thousand inhabitants in the country districts, as above written.

Again, by reference to the aggregation of the vital statistics of the principal cities of the United States as taken from the United States census reports, 1889–90, we find that less than one-half of one per cent. of all deaths therein reported were caused by appendicitis. But this comparative infrequency of deaths from appendicitis is not exceptional nor peculiar to the country districts and cities of the United States. Mulhall, in his tabulation of the vital statistics of European countries (1896), does not mention any deaths from appendicitis at all.

From these necrological data as culled from different sources, we find that the percentage of deaths occurring from appendicitis, in the great cities as well as in the country districts, is exceeding small as compared with deaths occurring from other inflammatory diseases involving organs whose textural anatomy is not altogether dissimilar to the anatomic structure of the vermiform appendix.

Therefore, from a consideration of my own experience and from the experience of those of my medical brethren whose duties like my own abound in the country, and from a review of the statistics of the great cities, as above written, I am led to the conclusion that notwithstanding the appendix is a blind wormlike tube, and having its open end situated near the base of the cecum, and therefore supposed to be liable to become the receptacle of numerous and variable indigestible ingesta which enter with food substances, and otherwise, the alimentary canal, yet it is a fact that it is not more subject to inflammatory or other diseased action than are other organs whose anatomic structure is of similar component parts.

These are interesting facts and deserve more than a passing notice.

Therefore, while studying the appendix, its shape, its position and its relation to the rest of the alimentary economy, in connection with the above written facts, we are prompted to ask, why does this marked freedom from inflammatory disease with regard to this peculiar organ exist? There must be a cause for it. There is a cause for everything and for every condition that exists, and I am persuaded that the cause for the very infrequent occurrence of appendicitis may be found by a study of its textural anatomy and of its relation to both the ileum and the cecum, and of its physiological anatomy.

By a painstaking study of these the reason opens up to us, and the answer being based upon philosophic principles, is not difficult, and may be formulated thus: Because of its anatomic and physiologic adaptability and ability to avoid and resist etiologic factors in the production of disease.

I am not of those who believe that the appendix is a veritable death-trap. I am not of those who say that it is rudimentary, nor of those who suppose that it is functionless and useless.

Because I am unable to explain its use and because I may not be acquainted with its function, I am not willing to say that it possesses neither; and because I find it just where it is with regard to its relation, shape and position, I am not ready to declare that it is rudimentary. I do not believe that it is a remainder of what was once more prominently developed, and what was once of more importance to the human economy than it is at present, and because of gradual degeneration or retrogressive growth, or involution, has attained to its present condition and contour. I do not believe that it is the result of so-called evolution, or the result of environment.

Indeed, I am not sure that any animal structure, nor any animal is such because of evolution or environment. It seems unreasonable to me to regard the human being in all its parts, organs and functions, wonderful and complex as they are in their almost perfect mechanism, as the result of unguided, undirected evolution from inanimate, senseless matter.

When I view my "Crescent street" movement, existing in all its mechanical delicacy, beauty and accuracy—not varying one minute in a year—I cannot look upon it and imagine that it exists because certain inert, senseless metals, so selected, arranged and modified themselves because of their environment, and because of necessity, as to develop naturally into the wonderful time-keeping machine that it is. On the contrary, we know that it is the result of the labor of trained workmen, working under the direction of a master workman, guided by plans evolved in the mind of an intelligent thinker. So when I view the human eye, far more delicate, complex and perfect in its vital completeness than any watch, I cannot look upon it and imagine that it exists because certain inorganic, lifeless particles of matter so selected, modified and arranged themselves, because of their environment and because of necessity, as to develop, undirected, into the glorious organ that it is. On the contrary, I am persuaded that it is the workmanship of a Supreme Intelligence, and wrought according to design, and for a purpose.

So also the vermiform process, while it cannot be compared to the eye in astuteness of anatomy, nor in completeness of function, nor in perfection of usefulness, yet, while I am not altogether able to give a reason for the faith that is in me, I am nevertheless persuaded that it has both a function and a use; and, the fact that a man lives and enjoys apparently as good health after having had his appendix removed as before its removal, is not a positive and conclusive argument that the said appendix is useless. There have been, and there are at present, men who have had portions of their intestinal tube removed, and yet live and enjoy apparently as perfect health as before its removal.

I cannot think the wonderful Designer, the Supreme Intelligence, whose works are all wrought in wisdom, would so hang a functionless, useless part of creation, sack-like, mouth-open, in such a place and in such a position as that it would invite every passing substance to drop in and set up a condition which would be likely to destroy the whole of the other parts of the said creation. Hence, I cannot believe that the appendix remains where it is as an accident of evolution, but that it is there to fulfill some purpose, and has a function, and is not useless, and by no means a veritable "death-trap," as declared to be by some.

And while we may not be able to explain its use nor define its function, yet a careful study of its homological, textural and physiological anatomy enables us to find out why it is—as we have seen—so well adapted to avoid and resist etiologic factors in the production of disease.

In noticing, first, its position in its relation to both the cecum and the ileum, we observe that it is not suspended from the very bottom or most dependent part of the cecum, dangling from it like a cord suspended from the ceiling, but arising from the fundus of the cecum at the juncture of the longitudinal muscular bands which course over the cecum and head of the colon, which point of juncture is a little above and behind the real bas-fond of the cecal pouch, it is directed upward, inward and behind the cecum, and in some instances with its terminal or closed end lying up beside the posterior wall of the ileum, and held in that position by a mesentery. I have found this to be the case in every instance where I have examined the appendix in making autopsies upon the bodies of persons dying of other diseases.

Lying thus in the same direction, and having its lumen somewhat parallel with that of the ileum, its open end, therefore, is considerably lower—when the man is sitting or standing—than its closed end, and it could well be compared to a small, somewhat crooked or bent test-tube, inverted, not standing upright but leaning, having an inclination of sixty to twenty degrees above the horizontal plane.

Entering the cecum and opening into it through the same side and from the same direction as does the ileum, a little below the ileo-cecal valve, any substance passing from the ileum through its valve passes directly over the orifice or open end of the appendix, but on account of the natural law of gravity does not turn backward and upward into its lumen.

Again, the contraction of the longitudinal muscular bands which pass over the cecum, contracting that organ in order to force its contents out and up into the ascending colon, contracting first at the point of their juncture, closes the orifice of the appendix before the contraction of the whole organ takes place; thus any irritating or other substance which might be forced back into the lumen of the appendix by the systaltic shrinkage of the cecum is prevented.

This, I believe, is the main reason why numberless seeds of various kinds, and other irritating substances foreign to the intestinal tract, do not find their way into the lumen or body of the appendix.

Again, the muscular membranes of the vermiform process, like those of other parts of the intestinal tube, are both longitudinal and transverse or annular. Their function is to shorten its length and diminish its caliber. Hence, it is capable of peristaltic or wormlike contraction in like manner as are other portions of the intestinal tube. Its mucous membrane is capable of secreting and does secrete mucus in abundance, so if by any means a seed or a number of seeds, or any rough or irritating substance should find its way into its lumen, it is admirably prepared first to protect itself against the said irritant by covering it thoroughly with mucus, and then to rid itself of the offending matter by belching it out into the cecum again by means of its peristaltic contraction.

This physiologic act is somewhat similar to the action of the eye in protecting and defending itself against any particle of dust, gravel, small insect or other irritant that might fall or be driven upon its sclerotic or corneal surface. So also is the act of sneezing when an irritant rests upon the pituitary membrane.

There are causes, however, which may produce appendicitis other than that of the lodgment of a foreign body in its lumen. It may become affected by a secondary inflammation or by an inflammation extending to it which had its origin in the cecum, or in other parts of the colon, or in the small intestines, such as ileocolitis, typhoid fever and such like diseases. Again, it is said that appendicitis may be caused by a traumatism from a blow or other force exerted upon the abdominal parietes.

It is not proof against nor exempt from catarrhal inflammation; indeed I am inclined to think that the most common cause of all appendicular disease is of so-called catarrhal character; and I can well understand how a continued inflammatory or non-inflammatory catarrhal condition could ultimately result in stenosis of the orifice, and possibly of the entire lumen of the appendix, by producing hypertrophy of its mucous and muscular membranes; and should such a narrowing of its orifice obtain I can well understand how disease of a very grave character might at any time ensue.

But here again nature has arranged for its protection against an active inflammatory process by refusing to supply it with a highly organized arterial system; depletion being one of the most powerful natural agencies in preventing and abating the inflammatory process in any organ.

In this connection it may be well to suggest that not every case of suspected appendicitis is such in reality. I have known of a number of cases of pelvic cellulitis or parametritis to have been mistaken for appendicitis; and in two instances nephritic colic, on account of its severity, and because the right ureter was involved, was supposed, at first, to be well-developed cases of appendicitis.

In November, 1886, I was asked to see a case of appendicitis in consultation with an eminent physician of an adjoining neighborhood. The case was that of a robust negro, middle-aged, who had been attacked four or five days before with a very severe pain in the lower portion of the right iliac region. The pain was incessant, and several rigors followed each other at irregular intervals during the first two or three days, and fever of a remittent type was present continuously.

None of the symptoms having abated, I was asked to see the case, with a view to operative interference, on the fifth day. Before I arrived at the negro's house I learned that he was dead. Having obtained the assent of his family, we made a *post-mortem* exploration the following day. The autopsy proved the diagnosis to be altogether incorrect—the appendix being intact and apparently free from disease of any kind. The negro had died of strangulated inguinal hernia. A very small knuckle of intestine had been forced by some means beneath Gimbernat's ligament and had been held there, undetected by the physician, sufficiently well to produce strangulation, inflammation, necrosis and dissolution.

Appendicitis, however, does occur, more frequently in the cities, less frequently in the country; and when a case has fully developed, or when it has not yet fully developed, but suspected of being such, it demands the most thoughtful and careful attention; and when a case has fully developed to the extent of unmistakable diagnosis, no clinician, however eminent he may be, can determine at once whether it belongs to the domain of medicine or to that of surgery; and to assert that every case belongs exclusively to the domain of either science is to assume what no man can establish.

I have not found it necessary to operate upon any of the five cases which have fallen into my hands—every case having yielded promptly to the depleting influence of small doses of the sulphate of magnesia frequently repeated. I agree, however, that it would be quite unreasonable to contend that every other case would yield as readily to the same plan of treatment.

Should any hard substance, angular, spiculated or otherwise rough enough to produce irritation, enter the body of the appendix, or should an enterolith form within its lumen which could not be thrown out by systaltic contraction, or should stenosis exist, especially about its open end, and should acute suppurative inflammation ensue, then, it seems to me, operative interference would be imperative. I would as soon expect such a case to get well without surgical aid as that a case of cystitis could be cured by medicines while an irritating calculus remained within the walls of the urinary bladder. Again, it seems to me that it would be just as unwise and unphilosophic to contend for an operation upon every case of recent catarrhal appendicitis. While it would be wise to interpose surgical aid in a given case of empyemic or suppurative pneumonitis, no one would think of using the knife for every case of simple lobar or catarrhal pneumonia. The inference is easy: All inflammations do not result in suppuration, and those inflammatory processes which tend to suppuration may be inhibited by proper and prompt means.

In conclusion: In a recent acute appendicitis I usually commence with one or two grains of calomel, and repeat every hour until three or four doses have been given; then follow with sulphate of magnesia 60 grains and bicarbonate of soda 10 grains, given in half a glass of chloroform water, and repeated every half hour until free catharsis is established; and if, in the meantime, pain is unbearable, I resort to the alleviating and soothing influence of chloroform given from time to time by inhalation. In this manner I have kept my patients from suffering and have brought them safely through in every instance.

I have found this plan of treating appendicitis admirably adapted to pelvic and other forms of peritonitis.

